# Islet-intrinsic effects of *CFTR* mutation

**DOI:** 10.1007/s00125-016-3936-1

**Published:** 2016-03-31

**Authors:** Fiona N. Manderson Koivula, Neville H. McClenaghan, Alan G. S. Harper, Catriona Kelly

**Affiliations:** 1 Northern Ireland Centre for Stratified Medicine, University of Ulster, C-TRIC, Altnagelvin Hospital Site, Glenshane Road, Derry/Londonderry, BT47 6SB Northern Ireland UK; 2 School of Biomedical Sciences, University of Ulster, Coleraine, Northern Ireland UK; 3 Institute for Science and Technology in Medicine, Keele University, Guy Hilton Research Centre, Stoke-on-Trent, UK

**Keywords:** Beta cells, CFTR, Cystic fibrosis, Diabetes, Endocrine, Review

## Abstract

Cystic fibrosis-related diabetes (CFRD) is the most significant extra-pulmonary comorbidity in cystic fibrosis (CF) patients, and accelerates lung decline. In addition to the traditional view that CFRD is a consequence of fibrotic destruction of the pancreas as a whole, emerging evidence may implicate a role for cystic fibrosis transmembrane-conductance regulator (CFTR) in the regulation of insulin secretion from the pancreatic islet. Impaired first-phase insulin responses and glucose homeostasis have also been reported in CF patients. CFTR expression in both human and mouse beta cells has been confirmed, and recent studies have shown differences in endocrine pancreatic morphology from birth in CF. Recent experimental evidence suggests that functional CFTR channels are required for insulin exocytosis and the regulation of membrane potential in the pancreatic beta cell, which may account for the impairments in insulin secretion observed in many CF patients. These novel insights suggest that the pathogenesis of CFRD is more complicated than originally thought, with implications for diabetes treatment and screening in the CF population. This review summarises recent emerging evidence in support of a primary role for endocrine pancreatic dysfunction in the development of CFRD.

**Table:** 

Summary
• CF is an autosomal recessive disorder caused by mutations in the *CFTR* gene
• The vast majority of morbidity and mortality in CF results from lung disease. However CFRD is the largest extra-pulmonary co-morbidity and rapidly accelerates lung decline
• Recent experimental evidence shows that functional CFTR channels are required for normal patterns of first phase insulin secretion from the pancreatic beta cell
• Current clinical recommendations suggest that insulin is more effective than oral glucose-lowering drugs for the treatment of CFRD. However, the emergence of CFTR corrector and potentiator drugs may offer a personalised approach to treating diabetes in the CF population

## Introduction

Cystic fibrosis (CF) is the most common autosomal recessive disorder in white people, and results from mutations in the cystic fibrosis transmembrane-conductance regulator (*CFTR*) gene, located on the long arm of chromosome 7 in humans [1]. CFTR is an apical membrane Cl^−^ channel that controls epithelial fluid and salt secretion, where mutations in *CFTR* lead to dehydrated, acidic secretions, which drive CF disease [2].

**Fig. 1:**
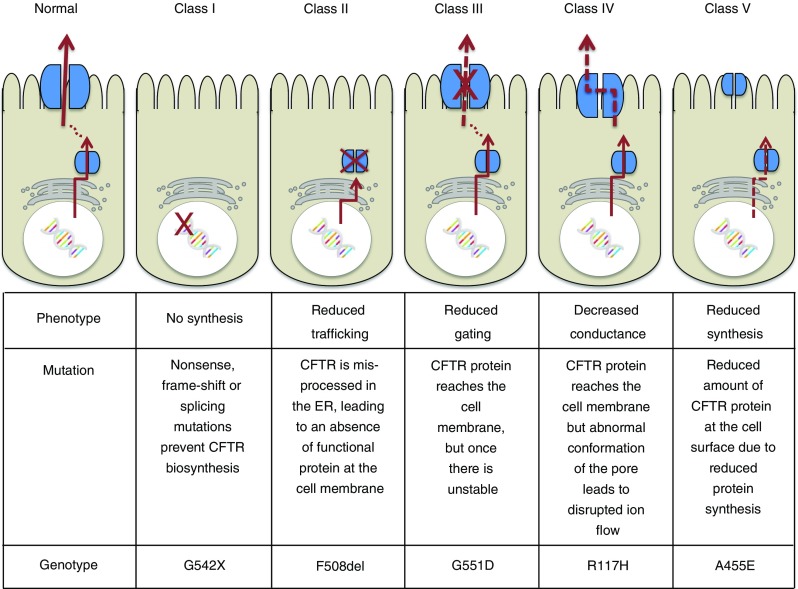
Classification of *CFTR* mutations. Approximately 1,000 *CFTR* mutations have now been identified. In the region of 20 of these mutations are thought to be disease causing and can be classified according to the resulting effect on CFTR protein production. Broadly speaking, class I mutations are associated with more severe phenotypes than class V mutations, although in CF, as with other complex genetic conditions, genotype does not always predict phenotype

*CFTR* is highly expressed in the intestines, pancreas, lungs, sweat glands and kidneys. The CFTR protein is a 1,480-amino-acid structure, consisting of two homologous halves, with each half consisting of six membrane-spanning segments and a nuclear binding domain (NBD) [3]. Like other integral membrane proteins, CFTR is synthesised in the endoplasmic reticulum (ER) and moves to the Golgi before being trafficked to the apical membrane [4]. Approximately 77% of the protein resides in the cytoplasm, 19% spanning the membrane and 4% in an extracellular loop [5]. Of the thousand or so *CFTR* mutations that have been identified, approximately 20 are understood to be disease causing and are categorised into five classes of mutations of increasing disease severity, as summarised in Fig. 1. The most commonly reported mutation results from a phenylalanine deletion at position 508 (F508del), with at least one allelic copy of this mutation present in 70–90% of patients with CF [2]. Class II mutations, including F508del, result from misprocessing of CFTR in the ER, leading to an absence of functional protein at the plasma membrane. Class III mutations, such as G551D (which is reported in approximately 5% of CF patients), are correctly processed and trafficked to the plasma membrane, but lack stability at the apical membrane [6].

## Clinical implications of *CFTR* mutation

Lung disease is the primary cause of morbidity and mortality among CF patients and results from recurrent and chronic bacterial infection. However, CF-related diabetes (CFRD) is the most common extra-pulmonary comorbidity, with patients presenting with worsened pulmonary function, a greater frequency and severity of pulmonary exacerbations and a greater prevalence of bacteria in the sputum [7]. In CF patients, pulmonary exacerbations usually result from bacterial or viral infections, which are often associated with cough and increased morbidity for the patient. Culturing *Pseudomonas aeruginosa* (the primary colonising bacterium in the CF lung) on medium containing glucose at levels found in CF airways (~59% of systemic levels) rather than glucose levels found in the airways of healthy individuals (~10% of systemic levels) results in a significant increase in bacterial proliferation [8].

While diabetes is a systemic condition affecting many organs, the lung is not usually considered an end target of the disease. However, a community-based cross-sectional study of 11,262 adults, 1,100 of whom had type 2 diabetes and none of whom had a diagnosis of any chronic lung disease, discovered that diabetes was associated with a restrictive defect and a 2–4% decrease in lung function [9]. Consistent with this, a prospective study of 4,434 men concluded that restrictive lung defects were associated with type 2 diabetes [10].

Clinically, CF patients diagnosed with CFRD have a six times greater risk of premature death compared with CF patients without diabetes [1]. Current estimates suggest that CFRD is present in approximately 2% of children, 19% of adolescents and 50% of adults with CF [11]. Moreover, the rapidly increasing incidence in recent years [11] may be attributed to enhanced screening programmes and/or advances in CF management. Although CFRD does not commonly present until adulthood, altered glucose homeostasis is often observed in childhood [12]. Oral glucose tolerance testing of 240 CF patients with and without overt diabetes demonstrated that patients with elevated glucose at 60 min had significantly reduced pulmonary function and increased HbA_1c_. In addition, patients with low plasma insulin at 60 min had significantly decreased pulmonary status and lower BMI. In all patients, pulmonary function was higher in patients with higher insulin levels at 60 min, irrespective of high or low glucose [13].

Patients with CFRD also have reduced height and weight [1] and chronically elevated protein catabolism compared with patients with CF and no diabetes. The elevation in protein catabolism may be successfully treated with exogenous insulin [1]. However, frequent illness, coupled with glucocorticoid use, means that insulin requirements in CFRD patients can be two or three times that of non-CF patients [14]. It has been proposed that loss of the anabolic effects of insulin can lead to subsequent protein catabolism which, in turn, causes clinical deterioration [14].

## Islet-intrinsic defects associated with *CFTR* mutation

The exact cause(s) of diabetes in CF patients remains unclear, although it is accepted that patients homozygous for F508del are most susceptible to developing CFRD [2], with suggestions that this may arise as a result of ER-stress-mediated apoptosis of the beta cell [15]. However, most CF patients demonstrate abnormalities in glycaemic control regardless of the class and severity of the *CFTR* mutation. Consistent with this, impaired first-phase insulin response has been reported in the absence of functional CFTR in both animal [16, 17] and human [18] studies.

*CFTR* mutations reduce bicarbonate-rich secretions from pancreatic ducts. This facilitates auto-digestion of the surrounding pancreatic tissue, leading to fibrosis and loss of both the exocrine and endocrine pancreatic tissue [19]. However, autopsy evidence from children and adolescents with CF suggests that abnormalities in beta cell size and shape can exist without any reported CFRD or exocrine fibrosis [20, 21]. Lombardo et al reported no change in exocrine pancreatic function in a group of CF patients over a 13 year study period, despite a significant increase in the prevalence of diabetes [22]. These clinical observations, coupled with recent functional studies in beta cell lines [23] and primary beta cells from mice [17] and humans [24], are consistent with the view that CFTR deficiency leads to islet-intrinsic defects in insulin secretion.

Several groups have reported CFTR expression [24–26] and small CFTR currents in human and mouse beta cells [24] and the RINmF5 beta cell line [26]. Pancreatic beta cells respond to a wide range of physiological and pharmacological modulators of insulin secretion, and many pathways controlling insulin secretion depend on beta cell ionic flux [27]. For example, a critical step in glucose-induced insulin secretion involves closure of ATP-sensitive K^+^ (K_ATP_) channels which, in turn, elicits membrane depolarisation and opening of voltage-gated Ca^2+^ channels. This rapid influx of Ca^2+^ then triggers a series of events leading to membrane fusion of insulin-containing secretory vesicles and insulin release [28]. ATP-sensitive K^+^ channels are also a target for insulin-releasing drugs, including the sulfonylureas [29]. Other nutrients also rely on pathways involving ionic flux to stimulate insulin release, including metabolisable and non-metabolisable amino acids, and amino acids co-transported with Na^+^ [30].

While most research to date has focussed on the role of cationic flux in beta cell function, much less attention has been directed to the movement of anions such as Cl^−^ [31]. In addition to the emerging role of CFTR, there are a number of Cl^−^ channels in the beta cell that are important for function. For example, C1C-3 is expressed on the secretory granule membrane and is implicated in the maintenance of granular pH. Inhibition of C1C-3 significantly decreased insulin granule exocytosis in mouse beta cells [32]. More recently, anotamin-1 (ANO1), a voltage-sensitive Ca^2+^-activated Cl^−^ channel, was found to play a role in glucose-induced insulin secretion from rat beta cells. Inhibition of ANO1 significantly reduced insulin release in response to glucose and resulted in a partial repolarisation of the beta cell [33].

Work in animal models has highlighted the potential of islet-intrinsic defects associated with CFTR deficiency or mutation. Evidence from newly established pig [34] and ferret [16] models suggest that the basic *CFTR* mutation has a pronounced effect on islet size and distribution in neonates. Olivier et al [16] reported reduced first-phase insulin secretion and abnormal glucose tolerance in fasted newborn *CFTR* ^−/−^ ferrets, a phenotype notably similar to CF human infants [16]. Stalvey et al [35] reported impaired glucose tolerance (consistent with observations in CFRD patients) in *CFTR* ^−/−^ mice exposed to streptozotocin. However, *CFTR* ^−/−^ mice that were not exposed to streptozotocin displayed normal glucose tolerance compared with controls.

**Fig. 2:**
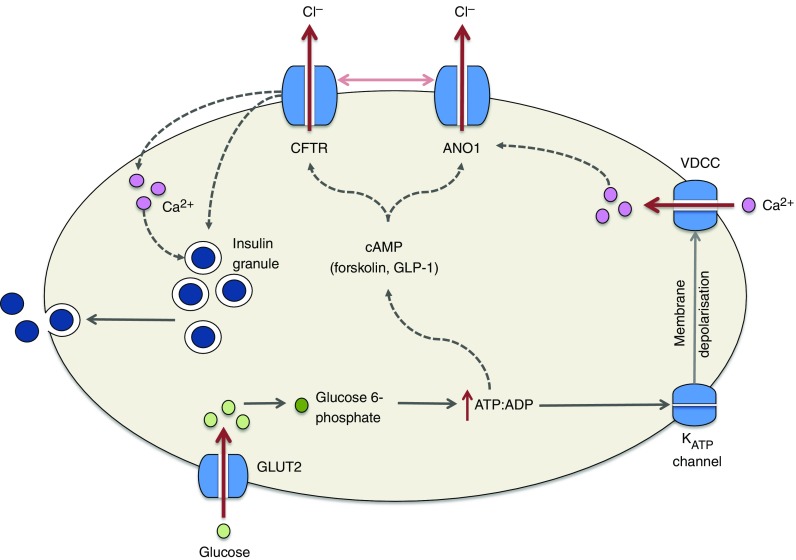
Potential mechanisms by which CFTR regulates insulin secretion from the beta cell. Glucose enters the beta cell through GLUT2 and is rapidly metabolised to glucose 6-phosphate, ultimately resulting in the generation of ATP, which causes the ATP-sensitive K_ATP_ channel to close. Membrane depolarisation and opening of voltage-dependent Ca^2+^ channels (VDCCs) ensue and calcium fluxes into the cell, resulting in insulin exocytosis. Recent studies have suggested that this process is hampered in the absence of CFTR, which may result from defects in ATP-generated cAMP activation of the CFTR channel. Indeed, pronounced reductions in insulin secretion are observed in response to forskolin- and GLP-1-stimulated increases in cAMP level. In addition, evidence suggests that CFTR (in conjunction with ANO1) may be involved in the priming of the insulin granule or in the regulation of calcium flux within the beta cell. The regulation of ANO1 by CFTR is denoted by the horizontal arrow between the two channels; dotted lines represent proposed mechanisms yet to be confirmed

In the past 2 years, experimental data from isolated primary beta cells, beta cell lines and transgenic animal models have identified potential mechanisms that surround the reduction in insulin secretion associated with CFTR deficiency (summarised in Fig. 2). Consistently, insulin secretion is reduced in the absence of functional CFTR channels as in, for example, studies using isolated beta cells exposed to CFTR inhibitors such as 3-[(3-triflouromethyl)phenyl]-5-[(4-carboxyphenyl)methylene]-2-thioxo-4-thiazolidinone (CFTR_Inh_-^172^) and CFTR inhibitor II (GlyH-101) [23, 24, 26, 36], studies in CFTR-deficient cell lines [23] and studies on islets from F508del mutant mice [26]. In primary human beta cells, insulin granule exocytosis in response to the cAMP agonists forskolin and glucagon-like peptide 1 (GLP-1) (which activate CFTR through the protein kinase A [PKA] pathway) was significantly decreased following treatment with CFTR_Inh_-^172^ and GlyH-101 [24]. On measurement of membrane depolarisations, this effect was most prominent in the initial depolarisations, which is in line with the decreased first-phase insulin secretion reported in CFRD patients [24]. This study also reported a novel role for CFTR as a regulator of ANO1. CFTR was shown to act upstream of ANO1, which played a contributory role in the regulation of insulin secretion from the beta cell, consistent with recent observations [33].

In addition to reductions in insulin secretion in response to cAMP activators, two independent studies have reported reductions in glucose-induced insulin secretion in models with CFTR interference [23, 26]. The CFTR inhibitor CFTR_Inh_-^172^ affected beta cell resting membrane potential and Ca^2+^ flux in RINmF5 cells [26]. MIN6 cells in which CFTR was silenced using short hairpin (sh)RNA also displayed a significant reduction in the ATP:ADP ratio, and this was associated with reduced glucose-induced insulin secretion [23]. These results suggest that the first-phase insulin response to glucose observed in CFRD patients may be partly attributable to responses mediated by membrane-bound voltage-dependent channels. Further investigations in an F508del mouse model confirm a significant reduction in glucose-induced insulin secretion in islets studied ex vivo. However, in contrast to the findings of the above studies, the authors concluded that the observed reduction in insulin secretion was directly proportional to the reduction in insulin content, and did not occur as a result of a CFTR-induced beta cell insulin secretory defect per se [17]. Despite a consistent reduction in insulin secretion, these preliminary studies merit the need for further mechanistic investigations into the role of CFTR in glucose-induced insulin secretion.

Given the dominant role of the insulin-secreting beta cell in diabetes pathogenesis, it is perhaps not surprising that beta cells have been the first target for preliminary investigations into the role of CFTR expression in CFRD. However, irregularities in circulating glucose can, of course, be a consequence of altered insulin, glucagon or somatostatin secretion [22]. While histological examination of autopsy material from patients with CFRD shows decreased beta cell mass [20, 21], the effect of the *CFTR* mutation on alpha and delta cells is less well studied [37]. Secretion of somatostatin appears to be preserved in CFRD [25]. Notably, CFTR expression has been reported in the alpha cells of the islets of Langerhans [37, 38] with few reports on CFTR localisation in delta cells. Many islet researchers studying ionic flux and hormone secretion in beta cells have begun to consider whether CFTR may impact on glucagon secretion. Glucagon is secreted by the alpha cells in response to hypoglycaemia, via processes inhibited by insulin and somatostatin [39]. Some evidence suggests that chronic hyperglycaemia may lead to decreased alpha cell activity [40], resulting in reduced islet glucagon secretion [41]. While impaired glucagon secretion is a common finding in CF [37], no difference in plasma glucagon was found between CF and non-CF fasted newborn pigs, despite impaired insulin secretion [34]. These initial observations prompt further investigations focussed on elucidating the effects of mutant CFTR on glucagon secretion and the alpha cell.

## Future directions and clinical implications

Sulfonylureas are often used as a first-line treatment in type 2 diabetes, as they increase insulin release from beta cells. Interestingly, sulfonylurea receptor 1 (SUR1), like CFTR, is a member of the ABC transporter superfamily of proteins, and shares significant homology with CFTR [29]. As such, compounds that open SUR1 and promote insulin transport may also act to decrease Cl^−^ transport in CFTR. Conversely, certain sulfonylureas may also increase the length of time that CFTR channels are in the activated state, thereby enhancing Cl^−^ movement across the cell membrane. In a study of 45 CFRD patients using either insulin or glibenclamide, no difference was found in forced expiratory volume in 1 s (FEV_1_), forced vital capacity (FVC) or weight to height ratio [42]. However, patients using sulfonylureas reported a better quality of life and decreased HbA_1c_ and their blood glucose levels were comparable with those of the insulin group. The efficacy of glibenclamide failed after an average of 18 months in these patients, who then commenced insulin therapy [42]. However, the first-line treatment for CFRD is usually insulin and, despite the apparent success of sulfonylurea treatment in the above study, current clinical guidelines suggest that insulin is more effective than oral glucose-lowering agents for the treatment of CFRD [43].

The development of CFTR correctors and potentiators offers great hope to CF patients and their families. These drugs act to correct the basic defect in CF and offer a personalised approach to the treatment of this debilitating disease. Currently, several corrector or potentiator treatments are licensed for the treatment of different *CFTR* mutations, including Ivacaftor, which targets the class III mutation G551D, and Lumacaftor, which targets the most common class II mutation, F508del. While the effect of these drugs on the progression of CFRD has been poorly studied to date, promisingly, a pilot study that investigated insulin function and the metabolic impact of Ivacaftor on CF patients carrying the G551D mutation reported improvements in glucose tolerance, with restoration of insulin responsiveness, particularly in younger participants [12].

While many aspects of CF pathology have been studied in detail and are well understood, it is only recently that attention has been directed towards the mechanisms underlying the association of diabetes with CF. Preliminary investigations have begun to unveil a potentially important role for pancreatic islet cells in the complex pathogenesis of CFRD, opening exciting new avenues of research with significant clinical potential. Future detailed studies focussing on the impact of CFTR in the regulation of endocrine pancreatic function offer great promise in understanding the fundamental molecular and metabolic basis and consequences of CFRD. These studies should directly impact on the therapeutic management of this hugely debilitating condition.

## Abbreviations

ANO1: Anoctamin 1
CF: Cystic fibrosis
CFRD: Cystic fibrosis-related diabetes
CFTR: Cystic fibrosis transmembrane-conductance regulator
CFTR_inh_^-172^: 3-[(3-triflouromethyl)phenyl]-5-[(4-carboxyphenyl)methylene]-2-thioxo-4-thiazolidinone
ER: Endoplasmic reticulum
GLP-1: Glucagon-like peptide 1
GlyH-101: CFTR inhibitor II
K_ATP_ channel: ATP-sensitive K^+^ channel
SUR1: Sulfonylurea receptor 1
